# Research on the synergistic effects of urbanization and ecological environment in the Chengdu–Chongqing urban agglomeration based on the Haken model

**DOI:** 10.1038/s41598-023-50607-1

**Published:** 2024-01-02

**Authors:** Weilong Wu, Ying Huang, Yuzhou Zhang, Bo Zhou

**Affiliations:** 1 School of Architecture and Environment, Sichuan University, Chengdu, 610065 Sichuan China; 2 Hubei Key Laboratory of Biological Resources Protection and Utilization, Hubei Minzu University, Enshi, 445000 Hubei China

**Keywords:** Ecology, Environmental sciences, Environmental social sciences

## Abstract

The development of urbanization and the protection of the ecological environment are important aspects of sustainable development in urban agglomerations; thus, their coordination and synergy are crucial. Scholars in both China and other countries have conducted extensive research on the relationship between urbanization and the ecological environment from the perspective of coordinated development. However, there is a lack of research on the synergistic effects of urbanization and the ecological environment. Taking the Chengdu–Chongqing urban agglomeration as an example, this study uses the Haken model to analyze the synergistic evolution mechanism, development patterns, and future trends of urbanization and the ecological environment. This study draws the following conclusions: (1) while a synergistic development mechanism has been established between urbanization and the ecological environment, the current system is still in a stage of low-level synergy, and the control system’s parameter for the direction and path of evolution is urbanization. (2) From 2001 to 2020, the level of synergistic development between urbanization and the ecological environment was relatively low, mainly moving from low-level synergy to medium–low-level synergy to medium-level synergy. (3) There is a significant spatial disparity in the levels of synergistic development, for which the leading region centers on Chengdu and Chongqing in the northwest and southeast, respectively, and the lagging region comprises the eastern part of the urban agglomeration, mainly represented by Dazhou, Kaizhou, and Yunyang. (4) The future trend of synergistic development between urbanization and the ecological environment in the Chengdu–Chongqing urban agglomeration is promising but requires increased infrastructure construction in mountainous urban areas, enhanced cooperation and circulation in transportation, logistics, information and other aspects, and balanced improvements in the level of synergistic development between urbanization and the ecological environment. The study of the synergistic development effect of urbanization and the ecological environment in urban agglomerations is of great significance for reducing the negative impact of urbanization on the ecological environment, increasing the positive interaction between the two, and promoting sustainable development in urban agglomerations.

## Introduction

Urbanization is one of the most important human activities on Earth^1^. Currently, global urbanization is showing signs of accelerated development. According to United Nations survey data, in 1950, only 30% of the global population lived in cities; however, by 2008, the urban population had exceeded 50%, and by 2050, this number is projected to reach 68.4%^2^. The rapid development of urbanization has changed people’s production and lifestyles and has also caused significant changes in regional environments, leading to various environmental issues^3–5^. In China, this phenomenon is even more pronounced. Since the twenty-first century, urbanization in China has made rapid progress, with an average annual growth rate of 1.04%^6^. Furthermore, China’s rapid urbanization has led to increasingly severe resource and ecological pressures, posing numerous sustainable development challenges for cities^7^, including air pollution^8^, traffic congestion^9^, energy shortages^10^, water quality deterioration^11^, ecosystem degradation^12^, forest/vegetation coverage^13^ and the loss or decline in biodiversity^14^. These issues are particularly prominent in expanding urban agglomerations. Urban agglomerations refer to spatial organizations that are compact and economically connected, are formed with one or more mega-cities or super-large cities as the core, and consist of multiple large cities as basic units that rely on a developed infrastructure network^15^. The main carrier of urbanization, urban agglomerations are gradually becoming a new territorial unit for national participation in global competition and the international division of labor; they are of great significance for national and regional economic development, international competitiveness, and sustainable development^16^. However, their rapid expansion and increasingly high density have led to some of the highest levels of urban diseases and ecological environmental problems. According to statistics, industrial wastewater emissions, industrial gas emissions, and industrial solid waste generation in Chinese urban agglomerations account for more than 67% of the national total. This means that although urban agglomerations contribute to over 75% of the national economic output, they also release over 75% of the pollutants nationwide^17^. In recent years, widespread smog has mainly been concentrated in the eastern coastal areas, northeastern regions, southwestern regions, and other areas where urban agglomerations are distributed, which fully reflects the increasing environmental pollution problems in urban agglomeration areas. Efforts to find solutions to this pressing issue have become important for academia and local governments. Therefore, it is crucial to strengthen research on the relationship between urbanization and the ecological environment in urban agglomeration areas.

Research on the relationship between urbanization and the ecological environment has mainly considered one-way influences, interactions, and coordinated development. Early research mainly focused on the one-way influence relationship between urbanization and the ecological environment, including the ecological changes caused by rapid urbanization and the constraints of the ecological environment on urbanization^18,19^. As research progressed, attention was given to the interaction between the two and derived theoretical models of the interaction, such as the Environmental Kuznets Curve (EKC) and the biexponential curve^20,21^. Quantitative empirical analysis of this interaction has been conducted using econometric models such as the decoupling model, VAR model, and GWR model^22,23^. In recent years, the coupling relationship and coordinated sustainable development of urbanization and the ecological environment have become research hotspots and frontier fields. International organizations have maintained a high level of attention to this topic and have listed it as a key research topic^24–26^. The academic community has also conducted theoretical and practical explorations. The Fang Chuanglin team has proposed the coupling circle theory, remote coupling theory, and the concept and research framework of the coupling cube to analyze the coupling and coordinated relationship between urbanization and the ecological environment^27–29^. Other scholars have used relevant mathematical models to quantitatively evaluate the coordinated development of the two, among which the most widely applied is the Coupling Coordination Degree Model (CCDM). Liao Chongbin^30^, Liu Yaobin^31^, and Qiao Biao^32^ successively established a coupling coordination model between urbanization and the ecological environment based on the coefficient of variation, coupling coefficient, and trigonometric function principles. The model has been highly regarded by scholars and widely used for practical research. For example, Huang Jinchuan and others used CCDM to analyze the relationship between urbanization and the ecological environment in Kazakhstan and combined it with geographic detectors to study the main control factors affecting coordinated development^33^. Wang Zhenbo and others focused on the Beijing-Tianjin-Hebei urban agglomeration, applied CCDM to analyze the relationship between urbanization and the ecological environment, and proposed a sustainable development path for green urbanization in the urban agglomeration^34^. Muhadaisi Ariken and others quantitatively evaluated the coordination relationship and spatiotemporal heterogeneity of urbanization and the ecological environment in provinces along the Silk Road in China through the integration of CCDM and GTWR models^35^. The above research has played an important role in understanding the evolutionary relationship and coordinated development of urbanization and the ecological environment. However, it cannot reveal the interaction process and evolutionary mechanism between these processes over time or explain the fundamental reasons for changes in the coordination relationship. Therefore, in addition to the traditional CCDM analysis method, an attempt is made in this study to find a new perspective and method to explore the evolutionary relationship between urbanization and the ecological environment. Hermann Haken’s synergetics theory has given us great inspiration. Synergetics theory states that subsystems and elements in a complex system will transform from disorder to order through internal interactions and mutual promotion, ultimately achieving the overall optimization and coordination of the system, that is, producing synergistic effects^36^. Haken’s model based on synergetics theory quantitatively analyzes the evolutionary mechanism of the coordinated development of complex systems by exploring the order parameters of their evolution; this method effectively explains the interaction process and degree of coordination between subsystems or elements^37^. Haken’s model has been widely applied in fields such as economics^38^, energy^39^, transportation^40^, and regional innovation^41^. However, it has rarely been applied to the relationship between urbanization and the ecological environment. Urbanization and the ecological environment gradually form an open complex system during the evolutionary process, and the two subsystems evolve and develop in a coordinated manner. Therefore, according to synergetics theory, Haken’s model can feasibly explore the synergistic interaction and evolutionary mechanism between the urbanization subsystem and the ecological environment subsystem, thus opening up a new path for future research on the relationship between urbanization and the ecological environment.

The Chengdu–Chongqing urban agglomeration is one of the five national-level urban agglomerations in China. It is an important demonstration area for the country’s promotion of new urbanization and undertakes the national strategic tasks of building the “fourth pole” of China’s future economic growth and the ecological security zone in the upper reaches of the Yangtze River. On January 3, 2020, General Secretary Xi Jinping made important instructions at the sixth meeting of the Central Committee for Financial and Economic Affairs, emphasizing the need to promote the construction of the Chengdu–Chongqing city cluster and create an important growth pole for high-quality development.

However, similar to other urban agglomerations, the Chengdu–Chongqing urban agglomeration faces severe ecological and environmental pressures while experiencing rapid economic and urbanization growth. Compared with other coastal urban agglomerations, such as Beijing–Tianjin–Hebei, the Yangtze River Delta, and the Greater Bay Area, the Chengdu–Chongqing urban agglomeration has more complex topography, climate conditions, and a fragile ecological environment, which frequently leads to natural disasters and serious soil erosion^42^. The industrial density in the Chengdu–Chongqing urban agglomeration is high, and the basin topography makes it difficult for industrial pollutants to dilute and diffuse, often resulting in severe regional air pollution^43^. Furthermore, the expansion of urban construction land and the protection of arable land have led to substantial conflicts; serious pollution in some tributary water environments and overall unsatisfactory environmental quality have developed^44^. The two rounds of environmental inspections conducted by the central inspection teams in the Chengdu–Chongqing Urban Agglomeration from 2016 to 2021 also reflected these issues^45^. In addition, due to the low efficiency of water, land, and energy resource utilization in traditional manufacturing industries, the Chengdu–Chongqing urban agglomeration faces increasingly severe resource constraints and pressures. There is an increasingly imbalanced situation between urbanization development and ecological environment carrying capacity. It is urgent to explore the path of coordinated and sustainable development between urbanization and the ecological environment.

Based on the above background, this article takes the Chengdu–Chongqing urban agglomeration as a typical research area. First, the Haken model is used to analyze in detail the interactive process and evolutionary mechanism between urbanization and the ecological environment. Then, ArcGIS and spatial autocorrelation analysis are utilized to explore the spatiotemporal evolution pattern and spatial agglomeration characteristics of the coordinated development of urbanization and the ecological environment. Furthermore, the gray prediction model is used to infer the future trend of the coordinated evolution of urbanization and the ecological environment in the Chengdu–Chongqing urban agglomeration. Finally, feasible policy suggestions are proposed based on the research results. With the progress of urbanization and the continued growth of the urban population, more agglomeration areas, such as the Chengdu–Chongqing urban agglomeration, will emerge in the future; this study can serve as a typical demonstration.

## Materials and methods

### Study area and data sources

#### Study area

The Chengdu–Chongqing Urban Agglomeration (27°39′–109°03′ N, 101°56′–109°15′ E) is located in the southwestern part of China in the upstream region of the Yangtze River. It is one of the five major national-level urban agglomerations in China, centered on Chongqing and Chengdu. The region has diverse landforms, including plains, hills, and mountains, and is characterized by a typical basin landform. The climate is mainly a subtropical humid monsoon climate. The administrative scope of the Chengdu–Chongqing Urban Agglomeration includes 15 prefecture-level administrative units in Sichuan Province and 29 district/county-level administrative units in Chongqing, with a total area of 185,000 km^2^. The permanent population in 2020 was 98 million, accounting for 6.94% of the total population in the country. The regional GDP was 6.64 trillion yuan, accounting for 6.54% of the national total. The urbanization rate of the permanent population was 63.01%.

**Figure 1:**
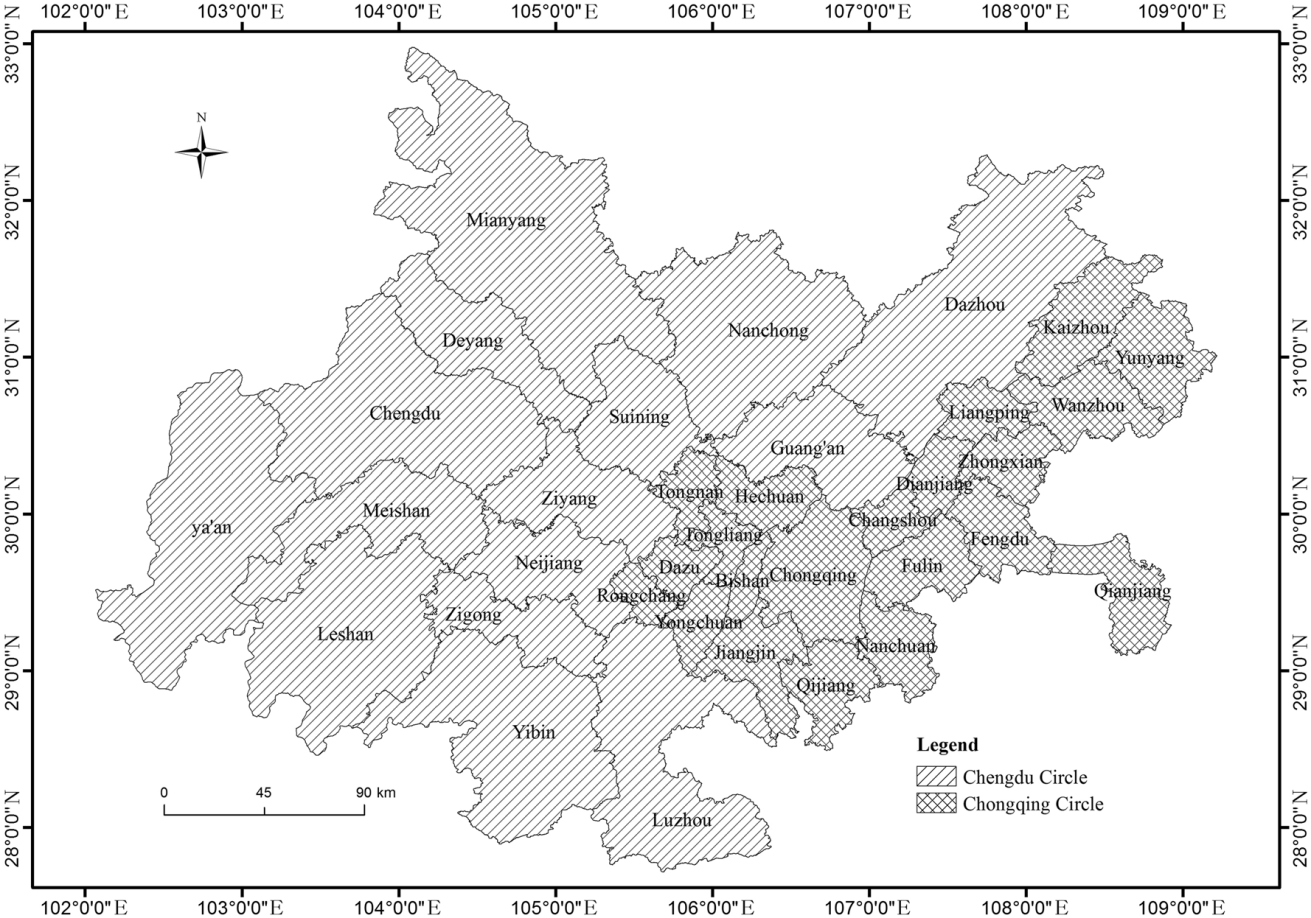
Scope of the Chengdu–Chongqing urban agglomeration (the image was created by the author using ArcGIS 10.6 software).

To meet the administrative characteristics and spatial analysis needs of Sichuan Province and Chongqing Municipality, the prefecture-level cities in Sichuan Province and the district/county-level administrative regions in Chongqing Municipality are considered units of the same nature and serve as the basic units for the research. For the sake of research spatial integrity and data availability, nine central urban areas in Chongqing, including Yuzhong District, were merged into one basic unit. Areas such as Mianyang, Dazhou, and Ya’an in Sichuan Province and Kaizhou and Yunyang in Chongqing, which are not within the scope of the Chengdu–Chongqing Urban Agglomeration, were included in the study. Finally, 36 basic research units were determined (Fig. 1).

### Data sources and preprocessing

The data used in this study include geospatial data and statistical data related to urbanization and the ecological environment. The geospatial data were obtained from the “Administrative Boundary Data of Chinese Counties” provided by the Resource and Environment Science Data Center of the Chinese Academy of Sciences (https://www.resdc.cn/Datalist1.aspx?FieldTyepID=5,2 accessed on March 5, 2023). The administrative boundary data containing 36 basic units were created through vector editing via ArcGIS 10.1 software. The statistical data on urbanization and ecological environment indicators were sourced from various publications, including the “China Urban Statistical Yearbook”, “China Urban and Rural Construction Statistical Yearbook”, “China County Statistical Yearbook”, “Sichuan Statistical Yearbook”, “Chongqing Statistical Yearbook”, “Sichuan Ecological Environment Bulletin”, and “Chongqing Ecological Environment Bulletin”, as well as city statistical yearbooks, national economic and social development statistical bulletins, and other relevant materials within the study area. The data cover the years 2001 to 2020. Missing data were supplemented and improved using interpolation and trend analysis methods. The statistical data involved were classified and organized using Excel software to create the Chengdu–Chongqing Urban Agglomeration Urbanization and Ecological Environment Database.

## Research methods

### Systematic index evaluation model

#### Construction of the indicator system

The urbanization and ecological environment system consists of two subsystems: urbanization and the ecological environment. Based on the connotation and development process of urbanization, an indicator system for assessing the status of the urbanization subsystem was constructed from four dimensions: Population, Economic, Sociology, and Space (PESS)^46^. Population urbanization serves as the foundation; economic urbanization represents the core content;, social urbanization reflects the diffusion of civilization and the level of people’s living standards; and spatial urbanization captures the changes in land use structure and the level of transportation infrastructure, which intuitively reflect the level of urbanization development. The pressure-state-response model (PSR) was used to construct an indicator system for assessing the status of the ecological environment subsystem; it represents the pressures faced by the ecological environment, the current characteristics of the ecological environment, and the response measures for the ecological environment^47^. The comprehensive assessment indicator system for the urbanization and ecological environment system is shown in Table 1.

**Table 1:** Assessment indicator system and weights for the urbanization and ecological environment system.

System	Subsystem	Primary indicator	Secondary indicator	Unit	Attribute	AHP method weights	Entropy method weights	Composite weights
Urbanization and ecological environment system	Urbanization system (UR)	Population urbanization	Permanent population urbanization rate	%	+	0.1564	0.0775	0.1162
			Proportion of employees in the secondary and tertiary industries	%	+	0.0777	0.0656	0.0756
		Spatial urbanization	Urban quantity density	Individual/hundred km^2^	+	0.0441	0.0582	0.053
			Urban area density	%	+	0.1293	0.0829	0.1096
			Transportation network density	km/ten thousand km^2^	+	0.0646	0.0858	0.0788
		Economic urbanization	Per capita GDP	Yuan	+	0.1128	0.0936	0.1083
			The proportion of value added from the secondary and tertiary industries to GDP	%	+	0.0757	0.0179	0.039
			Per capita social fixed asset investment	Yuan	+	0.0369	0.1257	0.071
			Per capita industrial output value	Yuan	+	0.0552	0.1302	0.0889
		Social urbanization	Per capita total retail sales of consumer goods	Yuan	+	0.0323	0.0719	0.051
			Per capita disposable income of urban residents	Yuan	+	0.0771	0.0442	0.0617
			Engel coefficient of urban residents	%	−	0.0221	0.0083	0.0143
			Number of cultural and artistic professionals per ten thousand people	People/ten thousand people	+	0.044	0.0611	0.0548
			Number of medical and technical personnel per ten thousand people	People/ten thousand people	+	0.0393	0.0351	0.0393
			Number of hospital beds per ten thousand people	Sheet/ten thousand people	+	0.0325	0.042	0.0385
	Ecological environment system (EG)	Ecological pressure	Average industrial wastewater discharge per unit of land area	Ton/km^2^	−	0.0929	0.1599	0.1307
			Average industrial gas emissions per unit of land area	Ten thousand cubic meters/km^2^	−	0.0302	0.0472	0.0405
			Average industrial solid waste generation per unit of land area	Ton/km^2^	−	0.1176	0.0344	0.0679
			Energy consumption per unit of GDP	Ton of standard coal/ten thousand yuan	−	0.0588	0.0258	0.0417
		Ecological conditions	Per capita water resources	m^3^	+	0.0494	0.0533	0.0538
			Per capita arable land area	hm^2^	+	0.0619	0.0426	0.0546
			Forest coverage rate	%	+	0.0991	0.047	0.0732
			Green coverage rate in built-up areas	%	+	0.0343	0.0153	0.0242
			Per capita park green space area in urban areas	m^2^	+	0.1123	0.0642	0.0911
		Ecological response	Urban domestic wastewater treatment rate	%	+	0.0518	0.1055	0.0784
			Urban domestic waste harmless treatment rate	%	+	0.0398	0.05	0.0478
			Industrial wastewater treatment compliance rate	%	+	0.0591	0.0206	0.0374
			Industrial exhaust gas removal rate	%	+	0.0734	0.0451	0.0617
			Comprehensive utilization rate of industrial solid waste	%	+	0.0271	0.0393	0.035
			Proportion of environmental governance investment to GDP	%	+	0.0923	0.2498	0.162

### Data standardization and weights

To eliminate the influence of differences in magnitude and dimensionality of each indicator on the calculation results, the indicators were standardized to reduce random interference. Different standardization formulas were used based on the attribute characteristics of the indicators:

1 $$ A_{ij} = \frac{{X_{ij} - \min (X_{ij} )}}{{\max (X_{ij} ) - \min (X_{ij} )}}{ (} \text{"} +\text{"} {\text{ indicator), }}A_{ij} \frac{{\max (X_{ij} ) - X_{ij} }}{{\max (X_{ij} ) - \min (X_{ij} )}} \, (\text{"} - \text{"} {\text{ indicator)}} $$

where *i* represents the index number; *j* represents the year; *X*_*ij*_ represents the actual calculated value; and max(*X*_*ij*_) and min(*X*_*ij*_) represent the maximum and minimum values of the *i*-th indicator, respectively. After standardization, larger values indicate better performance for all indicators.

A comprehensive weighted method that combines subjective and objective factors was used to assign weights to each indicator ^48^. First, the analytic hierarchy process (AHP) was used for subjective weighting, followed by the entropy method for objective weighting. Finally, the principle of minimum information entropy was utilized to calculate the comprehensive weights of both subjective and objective factors. The calculation formula is as follows ^48^:

2 $$ w_{i} = \frac{{\sqrt {w_{1i} \times w_{2i} } }}{{\sum\nolimits_{i = 1}^{n} {\sqrt {w_{1i} \times w_{2i} } } }} $$

### Calculation of the composite index

The composite index for both the urbanization and ecological environment subsystems was calculated using the linear weighting method. The calculation formula is as follows:

3 $$ f(x) = \sum\limits_{i = 1}^{n} {w_{i} } \times x_{i} , \, g{(}y{)} = \sum\limits_{j = 1}^{m} {w_{j} \times y_{j} } $$

where *f*(*x*) and *g*(*y*) represent the composite indices of the urbanization and ecological environment subsystems, respectively. *x*_*i*_ and *y*_*i*_ represent the standardized values of the indicators, while *w*_*i*_ and *w*_*j*_ represent the comprehensive weights of the indicators.

### Haken model

The Haken model, proposed by the founder of synergetic theory, Hermann Haken, is a valuable model for measuring the orderliness of a system. It evaluates the evolutionary stage of a system by identifying order parameters. Order parameters are parameters that dominate the macroscopic orderliness or patterns of the system. Based on the principles of order parameters, the complex self-organizing and cooperative evolution process of a system can be effectively studied. The main analysis process of the Haken model is as follows^49^:

#### Adiabatic approximation strategy

Assuming that the behavior of a system at a certain time *t*, denoted as *q*(*t*), depends solely on the external force *F*(*t*) at that time and decays over time, *F*(*t*) = *ae*^−*δt*^, where *a* is a constant and *δ* is the damping coefficient, the solution to $\dot{q}$(*t*) = *γq* + *F*(*t*) is:

4 $$ q(t) = \frac{a}{{\gamma { - }\delta }}(e^{ - \delta t} - e^{ - \gamma t} ) $$

Due to the instantaneous nature of the system’s response to external forces, there is no time for energy exchange to occur during this process. Therefore, this response process is referred to as an “adiabatic” process. Assuming that the rate at which the system’s behavior decays over time is much faster than the rate at which the external force decays over time, then:

5 $$ q(t) = \frac{a}{\gamma }e^{ - \delta t} = \frac{1}{\gamma }F(t) $$

The assumption *γ* ≫ *δ* is the prerequisite for using the adiabatic elimination method to eliminate fast variables. This principle is known as the adiabatic approximation principle.

#### Evolution equation of order parameters

Haken performed mathematical treatment on system parameters. Assuming *q*_1_ represents the internal force of a subsystem and *q*_2_ is controlled by this internal force, the system satisfies the following motion equation:

6 $$ \dot{q}_{1} = - \gamma_{1} q_{1} - aq_{1} q_{2} $$

7 $$ \dot{q}_{2} = - \gamma_{2} q_{2} + b\mathop q\nolimits_{1}^{2} $$

where *q*_1_ and *q*_2_ represent state variables, while *a*, *b*, *γ*_1_, and *γ*_2_ are control variables. The parameters *a* and *b* reflect the strength of the interaction between *q*_1_ and *q*_2_. γ_1_ and γ_2_ represent the damping coefficients of the two subsystems, with |*γ*_2_| ≥|*γ*_1_| (*γ*_2_ > 0) referred to as the “adiabatic approximation assumption” of the motion system. If the adiabatic approximation assumption holds true, removing *q*_2_ suddenly results in *q*_1_ not having enough time to change. By setting $\dot{q}_{2}$ = 0, we can obtain:

8 $$ q_{2} = \frac{b}{{\gamma_{2} }}\mathop q\nolimits_{1}^{2} $$

*q*_1_, which represents the order parameter, can be substituted into Eq. (8) to obtain the evolution equation of the order parameter:

9 $$ \dot{q}_{1} = - \gamma_{1} q_{1} - \frac{ab}{{\gamma_{2} }}\mathop q\nolimits_{1}^{3} $$

The above equation indicates that *q*_1_ determines *q*_2_, and *q*_2_ changes correspondingly with the variations in *q*_1_. Therefore, *q*_1_ is the order parameter of the system, governing and dominating the process of cooperative evolution in the system.

#### Potential function

Based on the system’s motion equation and the order parameter, the system’s potential function is determined to assess its state. Integrating the negative of $\dot{q}_{1}$ yields the potential function of the system, which effectively determines the overall state of the system:

10 $$ v = \frac{1}{2}\gamma_{1} \mathop q\nolimits_{1}^{2} + \frac{ab}{{4\gamma_{2} }}\mathop q\nolimits_{1}^{4} $$

Since the physical equations are formulated for continuous random variables, they need to be discretized when applied to urbanization and ecological environment analysis. This involves converting them into discrete form:

11 $$ q_{1} (t) = (1 - \gamma_{1} )q_{1} (t - 1) - aq_{1} (t - 1)q_{2} (t - 1) $$

12 $$ q_{2} (t) = (1 - \gamma_{2} )q_{2} (t - 1) + b\mathop q\nolimits_{1}^{2} (t - 1) $$

#### Degree of synergy

Based on the potential function, we can determine the stable point *A* of the system. The distance between any point *B* on the potential function and stable point *A* determines the system’s state, i.e., the level of synergy. We can calculate the distance between two points using a distance formula:

13 $$ d = \sqrt {\mathop {(x_{1} - x_{2} )}\nolimits^{2} + (y_{1} + y_{2} )^{2} } $$

The larger the value of *d* is, the less synergy there is in the system. Conversely, a higher value of *d* indicates a higher level of synergy in the system. To facilitate further analysis, we perform a reverse transformation on the d value by using the following formula:

14 $$ S = 1{ - }d $$

where *S* represents the degree of synergy.

### Spatial autocorrelation

Spatial autocorrelation effectively detects the spatial pattern characteristics of synergy development, including global and local spatial autocorrelation. Global spatial autocorrelation is usually represented by the *global Moran’s I* index and the *Getis-Ord general G* index. The former is used to test the similarity of spatial adjacency or proximity of research units, while the latter is used to further verify the existence of high or low value clusters in space. Local spatial autocorrelation is mainly measured by the Getis-Ord Gi* index, which can more accurately characterize the spatial differentiation characteristics of research units within a region. The formulas are as follows ^50^:

15 $$ Moran^{\prime}s \, I = \sum\limits_{i = 1}^{n} {\sum\limits_{j = 1}^{n} {W_{ij} (x_{i} - \overline{x})(x_{j} - \overline{x})} } /s^{2} \sum\limits_{i = 1}^{n} {\sum\limits_{j = 1}^{n} {W_{ij} } } $$

16 $$ G(d) = \sum\limits_{i = 1}^{n} {\sum\limits_{j = 1}^{n} {W_{ij} x_{i} x_{j} } } /\sum\limits_{i = 1}^{n} {\sum\limits_{j = 1}^{n} {x_{i} x_{j} } } $$

17 $$ G_{i}^{*} (d) = \sum\limits_{j = 1}^{n} {W_{ij} x_{j} } /\sum\limits_{j = 1}^{n} {x_{j} } $$

where $\overline{x}$ and *s*_2_ represent the mean and standard deviation of variable *x*, respectively. *n* is the number of research units. *x*_*i*_ and *x*_*j*_ are the attribute values of spatial units *i* and *j*, respectively. *W*_*ij*_ is the spatial weight matrix.

### Gray prediction model

Gray system theory focuses on the study of “small sample” and “poor information” uncertainty systems, where only partial information is known, while the rest remain unknown. By generating and developing the known information, valuable insights are extracted to accurately describe and effectively monitor the system’s operational behavior and evolutionary patterns. In this study, the Gray Prediction GM (1, 1) model is employed to quantitatively predict the evolution of synergy over time and obtain future values of this indicator. The main analytical process of the model is outlined as follows^48^:

First, let us assume that the original time series is:

18 $$ A = [a_{0} (1) - \delta /\beta ]_{e}^{ - \beta k} + \delta /\beta , \, k = 1,2,...,n $$

By performing cumulative processing on *A*_0_, we generate the sequence:

19 $$ A_{1} = [a_{1} (1),a_{1} (2),...,a_{n} (n)] $$

Then, we calculate using a differential equation:

20 $$ \frac{{dA_{1} }}{dt} + \beta A_{1} = \delta $$

where *β* represents the development grayness and *δ* represents the endogenous control gray number. Let us assume the parameter vector to be estimated and solve the equation by using the least squares method. Finally, we obtain the prediction model as follows:

21 $$ a_{1}^{T} \overset{\lower0.5em\hbox{$\smash{\scriptscriptstyle\frown}$}}{A}_{1} (k + 1) = [a_{0} (1) - \delta /\beta ]_{e}^{ - \beta k} + \delta /\beta , \, k = 1,2,...,n $$

Finally, the effectiveness of the predictive model is validated before carrying out the prediction work. The mean absolute percentage error (MAPE) is an ideal indicator for measuring the prediction accuracy of the model^51^. The expression and evaluation criteria for MAPE are shown in Eq. (22) and Table 2^52^.

22 $$ MAPE = \frac{1}{n}\sum\limits_{k = 2}^{n} {\left| {\frac{{x^{(0)} (k) - \hat{x}^{(0)} (k)}}{{x^{(0)} (k)}}} \right|} \times 100\% $$

**Table 2:** MAPE criteria for model evaluation.

MAPE (%)	Prediction accuracy
< 10	Highly accurate predictability
10–20	Good predictability
20–50	Reasonable predictability
> 50	Weak and inaccurate predictability

## Results

### Mechanism of urbanization and ecological environment coevolution

#### Identification results of sequential parameters

The urbanization and ecological environment system consists of two variables, urbanization (UR) and ecological environment (EG). Based on the Haken model formula, an analysis is directly conducted on the constructed equations of these two variables. StataMP 17.0 software is used to regress panel data from 2001 to 2020, and the results indicate that UR is the sequential parameter for the coordinated development of the urbanization and ecological environment system, controlling the direction and path of system evolution (Table 3).

**Table 3:** Regression results of the Haken model.

Serial number	Model assumptions	Equations of motion	Control variables	Conclusion
①	*q*_1_ = UR *q*_2_ = EG	*q*_1_(t) = 1.1095***q_1_(t − 1) − 0.0791***q_1_(t − 1)*q*_2_(t − 1) *q*_2_(t) = 0.9669***q_2_(t − 1) − 0.0470***q_1_^2^(t − 1)	*γ*_1_ = −1095, *a* = 0.0791, *γ*_2_ = 0.0331, *b* = − 0.0470	(1) The equations of motion hold true (2) The adiabatic approximation assumption is satisfied (3) The model assumptions are valid, with urbanization as the serial parameter
②	*q*_1_ = EG *q*_2_ = UR	*q*_1_(t) = 1.0510***q_1_(t − 1) − 0.0900***q_1_(t − 1)*q*_2_(t − 1) *q*_2_(t) = 1.0259***q_2_(t − 1) + 0.0466***q_1_^2^(t − 1)	*γ*_1_ = − 0.0510, *a* = 0.0900, *γ*_2_ = − 0.0259, *b* = 0.0466	(1) The equations of motion are valid (2) The adiabatic approximation assumption is not met (3) The model assumptions are not valid

*, **, and *** indicate significance at the 10%, 5%, and 1% levels, respectively. No “*” indicates insignificance.

#### Coevolutionary characteristics

The equation of motion equation ① shows that *γ*_1_ = − 1095, *a* = 0.0791, *γ*_2_ = 0.0331, and *b* = − 0.0470. Therefore, the system evolution equation is:

22 $$ \dot{q}_{1} = 0.1095q_{1} + \frac{37}{{331}}\mathop q\nolimits_{1}^{3} $$

The system potential function is:

23 $$ v = { - }\frac{11}{{200}}\mathop q\nolimits_{1}^{2} { - }\frac{{{37}}}{1324}\mathop q\nolimits_{1}^{4} $$

**Figure 2:**
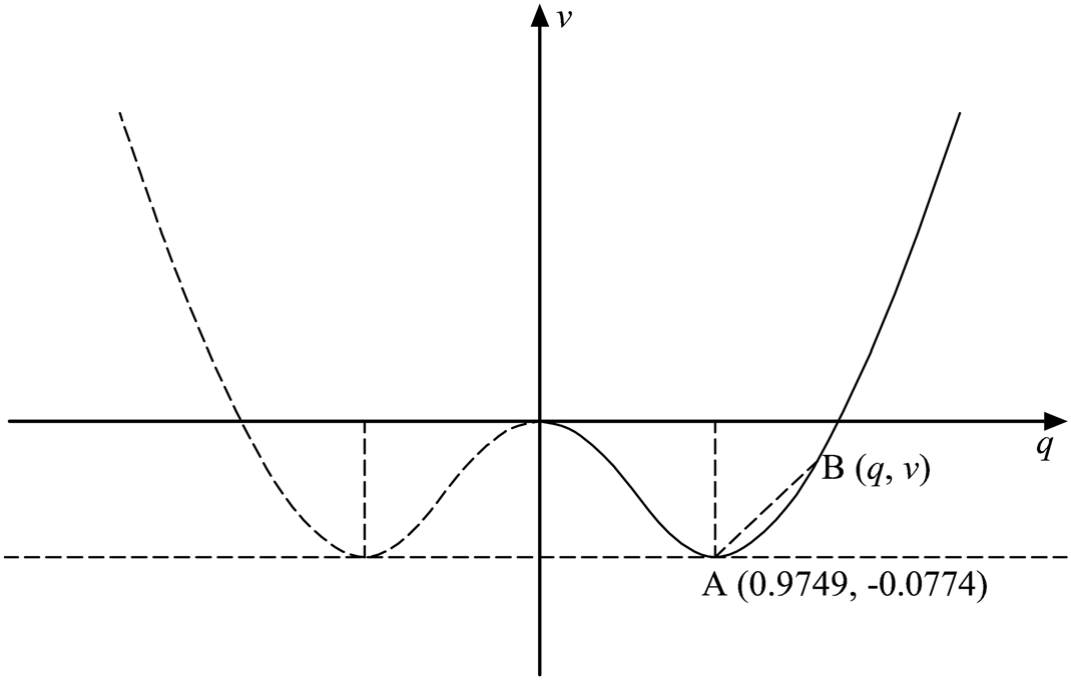
Potential function curve of the coordinated evolution of urbanization and the ecological environment system in the Chengdu–Chongqing urban agglomeration from 2001 to 2020 (the image was created by the author using AutoCAD 2008 software).

Let $\dot{q}_{1}$ = 0; then, the three solutions of the potential function are *q*_1_* = 0; *q*_1_** = 0.9749; *q*_1_*** = − 0.9749. Additionally, *v* = − 0.0774 is obtained. In the urbanization and ecological environment development system, the values of UR are all greater than zero, so the potential function graph considers only the part where *q* > 0. Based on the three solutions of the potential function and the value of *v*, the stable point of the system is determined as A(0.9749, −0.0774), and then the potential function graph (Fig. 2) is plotted. The potential function maps the evolution path of the system, and a slight change in the control parameter triggers drastic changes in other parameters, forming the “butterfly effect”, ultimately leading to a system mutation toward order.

The control variables (*a*, *b*, *γ*_1_, *γ*_2_) reflect the evolution behavior of the urbanization and ecological environment system. The variables *a* and *b* represent the synergistic effects of *q*_2_ on *q*_1_ and *q*_1_ on *q*_2_, respectively. When *a* is a positive value, *q*_2_ hinders the growth of *q*_1_, and the larger the absolute value of *a* is, the stronger the hindering effect. When *a* is a negative value, *q*_2_ promotes the growth of *q*_1_, and the larger the absolute value is, the stronger the promoting force. When *b* is a positive value, *q*_1_ promotes the growth of *q*_2_; when *b* is a negative value, *q*_1_ hinders the growth of *q*_2_. The variables *γ*_1_ and *γ*_2_ reflect the ordered states established by the system. When *γ*_1_ is negative, the q1 subsystem has established a positive feedback mechanism to promote the ordered evolution of the system. The larger the absolute value of *γ*_1_ is, the higher the degree of order. When *γ*_1_ is a positive value, the *γ*_1_ subsystem exhibits a negative feedback mechanism, and the larger the absolute value of *γ*_1_ is, the higher the degree of disorder, and the fluctuations of the system are amplified. When *γ*_2_ is a negative value, the *q*_2_ subsystem exhibits a positive feedback mechanism, which can enhance the degree of order in the system. When *γ*_2_ is a positive value, the *q*_2_ subsystem has established a negative feedback mechanism to enhance the degree of order.

The results of the motion equation show that *γ*_1_ = −1095, *a* = 0.0791, *γ*_2_ = 0.0331, and *b* = −0.0470. The values of control variables *a* and *b* indicate that the ecological environment has a negative impact on urbanization development, while urbanization development hinders the improvement of the ecological environment. The values of control variables *γ*_1_ and *γ*_2_ indicate that urbanization has established a positive feedback mechanism to promote the ordered evolution of the system, while the ecological environment subsystem has also established a negative feedback mechanism to enhance the degree of order. The absolute values of all four control variables are relatively small, indicating that the influence between the two subsystems is still relatively light, and the current system is in a stage of low-level order development.

### Synergistic evolution pattern of urbanization and ecological environment

Based on the results of the synergy calculation and referring to relevant research findings^41^, the synergy level of the Chengdu–Chongqing urban agglomeration is divided into five categories: low synergy type (0–0.2), medium–low synergy type (0.2–0.4), moderate synergy type (0.4–0.6), medium–high synergy type (0.6–0.8), and high synergy type (0.8–1).

#### Overall evolution characteristics

Selected for analysis are the synergistic development index for the years 2001 and 2020, as well as the average index from 2001 to 2020 (Table 4). Looking at the scale of the Chengdu–Chongqing urban agglomeration, the average synergistic development index for urbanization and the ecological environment from 2001 to 2020 is 0.3050, indicating a relatively low overall level of synergy. The synergistic development index shows a gradual increase, growing from 0.1419 in 2001 to 0.5236 in 2020, a 3.69-fold increase. At the interprovincial scale, the overall level of synergistic development in the Chongqing metropolitan area is higher than that in the Chengdu metropolitan area from 2001 to 2020. Initially, the synergy levels of both areas were relatively close, but after 2009, the synergy development speed in the Chongqing metropolitan area became significantly higher than that in the Chengdu metropolitan area. By 2020, the synergy development index between the two areas differed by 8.47 percentage points. At the city scale, the synergistic development levels of the 36 cities show gradual increasing trends but with significant variations. Most cities have transitioned from low synergy development types to moderate synergy development types, while Chengdu and Chongqing have reached a high level of synergy development.

**Table 4:** Synergistic level of urbanization and ecological environment in the Chengdu–Chongqing urban agglomeration from 2001 to 2020.

Chengdu metropolitan area	2001	2020	Average value^①^	Chongqing metropolitan area	2001	2020	Average value^①^
Chengdu	0.3282	0.8245	0.5584	Chongqing	0.3712	0.8419	0.6050
Zigong	0.1755	0.5161	0.3277	Wanzhou	0.1864	0.5320	0.3412
Luzhou	0.1239	0.4630	0.2582	Qianjiang	0.0968	0.5399	0.2685
Deyang	0.1969	0.5419	0.3453	Fulin	0.1940	0.6840	0.3992
Mianyang	0.1440	0.4633	0.2810	Changshou	0.1810	0.6652	0.3960
Suining	0.1356	0.4794	0.2769	Jiangjin	0.1712	0.5900	0.3415
Neijiang	0.1572	0.4625	0.2978	Hechuan	0.1619	0.5722	0.3312
Leshan	0.1329	0.4489	0.2706	Yongchuan	0.1989	0.6812	0.3984
Nanchong	0.1234	0.4553	0.2565	Nanchuan	0.1282	0.4945	0.2675
Meishan	0.0940	0.4104	0.2390	Qijiang	0.1167	0.5857	0.3069
Yibin	0.1136	0.4487	0.2602	Dazu	0.1120	0.6415	0.3178
Guang’an	0.0971	0.4522	0.2515	Bishan	0.1755	0.7200	0.3846
Dazhou	0.0961	0.3740	0.2040	Tongliang	0.1581	0.6778	0.3492
Ya’an	0.1147	0.3844	0.2356	Tongnan	0.1074	0.6103	0.2832
Ziyang	0.0920	0.3881	0.2405	Rongchang	0.1464	0.6778	0.3482
				Kaizhou	0.0782	0.4487	0.2175
				Liangping	0.1048	0.5242	0.2453
				Fengdu	0.0635	0.4821	0.2081
				Dianjiang	0.0922	0.5123	0.2520
				Zhongxian	0.0839	0.5045	0.2305
				Yunyang	0.0565	0.4661	0.1872
Average value^②^	0.1417	0.4742	0.2869	Average value^②^	0.1421	0.5929	0.3180

Average value^①^ refers to the 20-year average of the synergistic level of each city between 2001 and 2020. Average value^②^ refers to the average synergistic level of all cities in the Chengdu metropolitan area and Chongqing metropolitan area in 2001 and 2020, as well as the average synergistic level from 2001 to 2020.

#### Spatial distribution pattern

**Figure 3:**
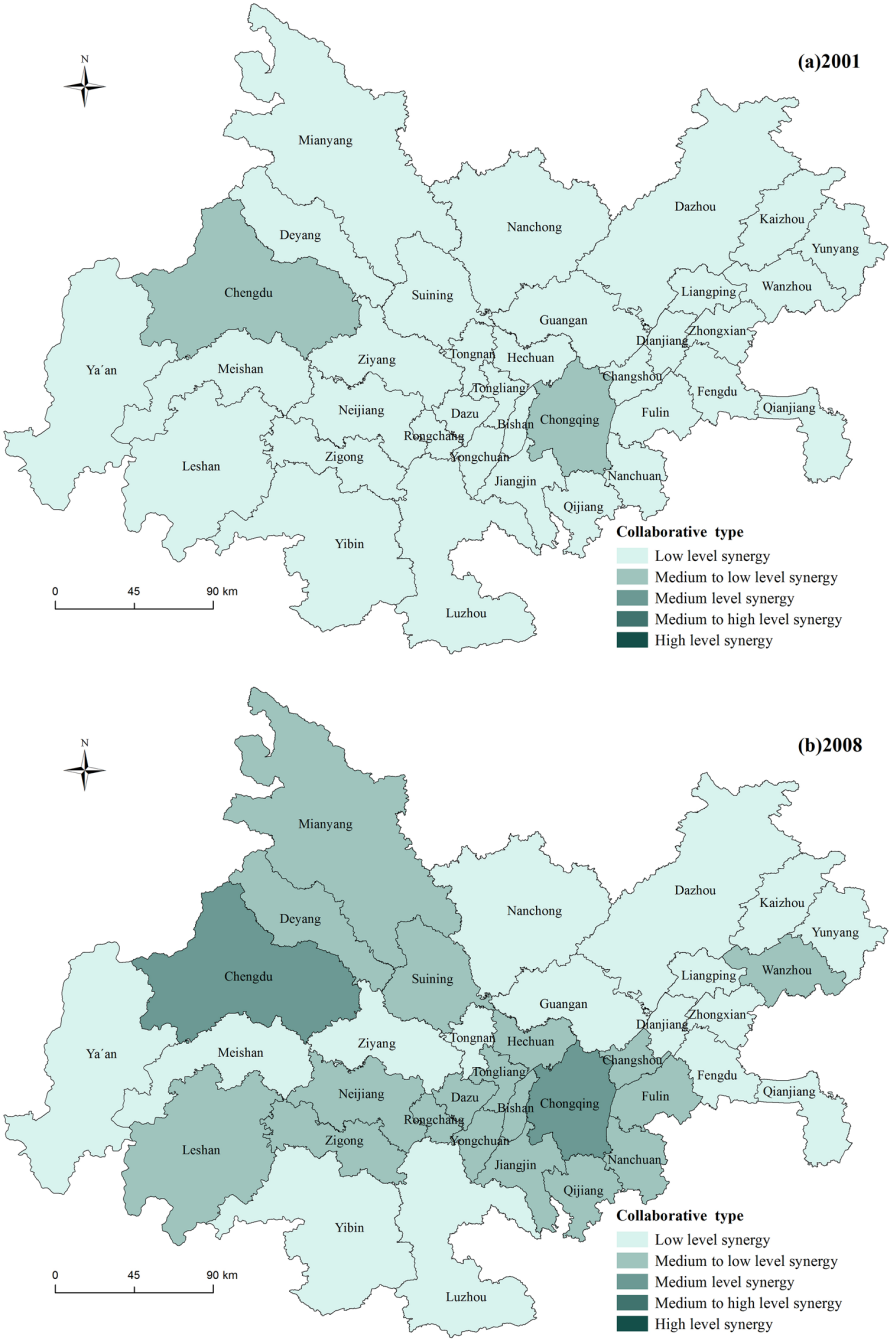

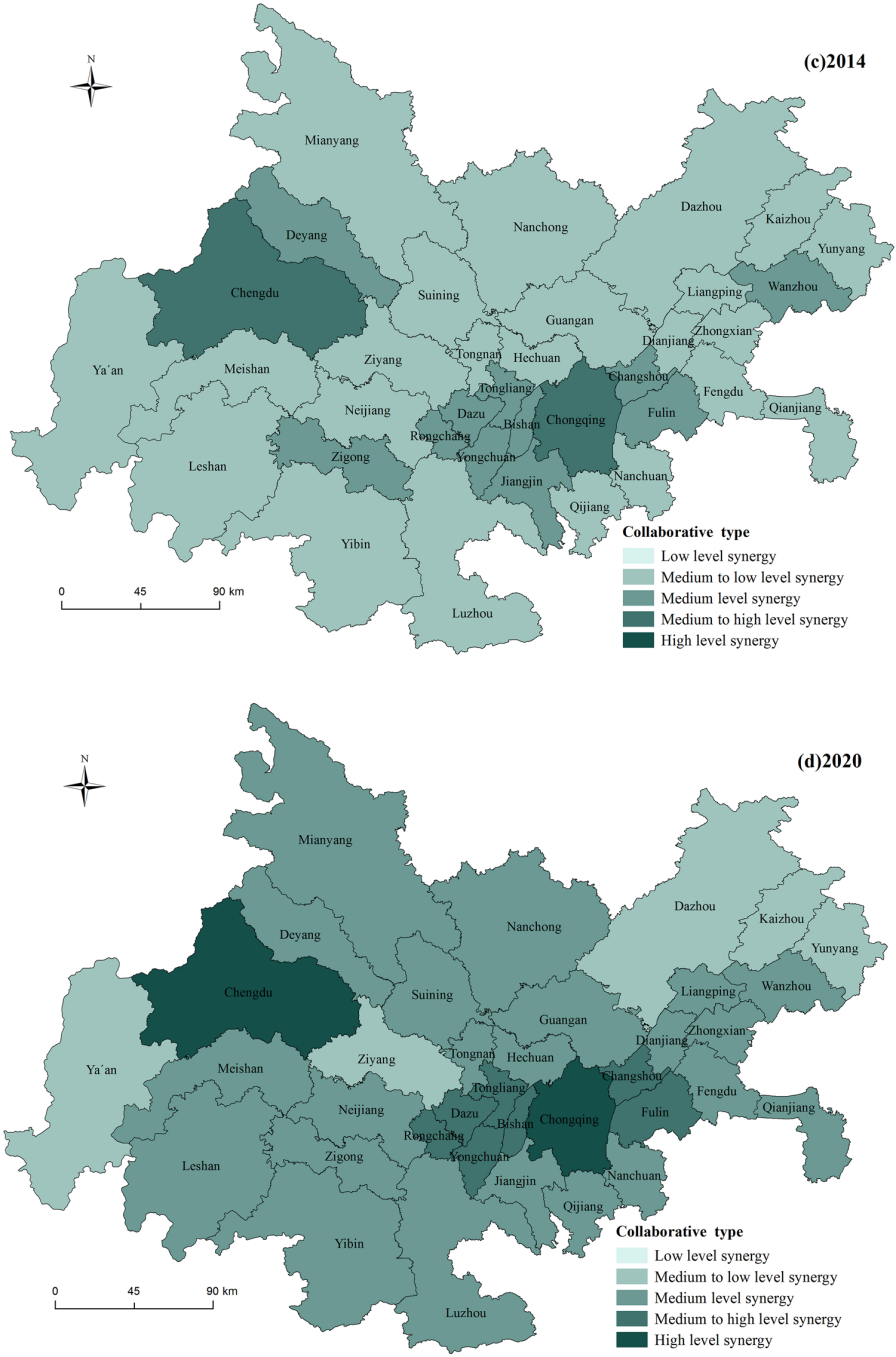
Spatial distribution of urbanization and ecological environment synergy level (the image was created by the author using ArcGIS 10.6 software).

The synergy development index for the years 2001, 2008, 2014, and 2020 were selected, and a spatial distribution map was created to analyze the spatial distribution characteristics of urbanization and ecological environment synergy development in the Chengdu–Chongqing urban agglomeration (Fig. 3). An analysis of Fig. 3 indicates that the synergy development level of urbanization and the ecological environment in the Chengdu–Chongqing urban agglomeration shows an upward trend. The synergy development types mainly experienced the stages of low synergy development, medium–low synergy development, and medium synergy development, with fewer instances of medium–high synergy and high synergy development types. The leading regions in terms of synergy development are the northwest and southeast regions centered on Chengdu and Chongqing, while the lagging regions in terms of synergy development are mainly located in the eastern part of the urban agglomeration, including Dazhou, Kaizhou, and Yunyang. (1) Low synergy development area: this is the main type of synergy in the initial stage of urban agglomeration development and represents the most widely distributed and largest proportion of synergy type throughout the period. In 2001, all regions except Chengdu and Chongqing were in the stage of low synergy development, and by 2008, 17 cities still exhibited low synergy characteristics. (2) Medium–low synergy development area: this is the longest-lasting type of distribution, observed in 2008, 2014, and 2020. Among these years, 2014 had the highest distribution, with 23 cities, and by 2020, five cities, Ya’an, Ziyang, Dazhou, Kaizhou, and Yunyang, were still in the stage of medium–low synergy development. (3) Medium synergy development area: the medium synergy development type started to appear in 2008, but only Chengdu and Chongqing were in this category. After 2014, this type gradually expanded outward from these two cities, and by 2020, the medium synergy development area had expanded to include 22 cities, becoming the main synergy type during this period. (4) Medium–high and high synergy development areas: the medium–high and high synergy development types were relatively rare between 2001 and 2020 and appeared only in 2014 and 2020. Apart from Chengdu, they were mainly concentrated around Chongqing. In 2014, only Chengdu and Chongqing exhibited medium–high synergy development characteristics, but by 2020, cities such as Fuling, Changshou, and Bishan around Chongqing had also joined this category, while Chengdu and Chongqing had evolved to the stage of high synergy development.

### Spatial clustering characteristics of urbanization and ecological environment synergy evolution

#### Global spatial autocorrelation

To further analyze the spatial clustering characteristics of urbanization and ecological environment synergy development in the Chengdu–Chongqing urban agglomeration, the *global Moran’s I* index and *Getis-Ord general G* index were calculated to examine the global spatial autocorrelation of the synergy development level (Table 5). From Table 5, the following observations can be made: (1) The *global Moran’s I* values for 2001 and 2008 are relatively small and do not pass the significance test, indicating that there is no spatial autocorrelation in the synergy development level of urbanization and the ecological environment in the Chengdu–Chongqing urban agglomeration between 2001 and 2008. However, the *global Moran’s I* values for 2014 and 2020 are 0.1172 and 0.2694, respectively, and they pass the significance test, indicating the presence of spatial autocorrelation in the synergy development level between 2014 and 2020. (2) The *global Moran’s I* values gradually increased from 2001 to 2020, with a significant increase observed between 2014 and 2020. This suggests that the spatial autocorrelation of the synergy development level of urbanization and the ecological environment is gradually strengthening over time. (3) The *G*(*d*) and *E*(*d*) values for 2008, 2014, and 2020 are similar and pass the significance test, indicating significant high-value and low-value clustering of urbanization and ecological environment synergy development during this period. However, the *P*(*d*) value for 2001 is 0.1310 and does not pass the significance test, suggesting that the high-value or low-value clustering characteristics of synergy development in 2001 are not significant. The differences between the *G*(*d*) and *E*(*d*) values for 2008, 2014, and 2020 are 0.0233, 0.0238, and 0.0292, respectively; this difference gradually increases, indicating an increasing trend of spatial clustering in the synergy development level between 2008 and 2020 in the Chengdu–Chongqing urban agglomeration.

**Table 5:** Global spatial autocorrelation of urbanization and ecological environment synergy level.

Year	*M*(*I*)	*Z*(*I*)	*P*(*I*)	*G*(*d*)	*E*(*d*)	*Z*(*d*)	*P*(*d*)
2001	0.0303	0.8190	0.4128	0.2118	0.1875	1.5103	0.1310
2008	0.0597	1.2217	0.2218	0.2108	0.1875	1.8064	0.0709
2014	0.1265	2.1139	0.0345	0.2113	0.1875	2.5100	0.0121
2020	0.2698	3.9575	0.0001	0.2167	0.1875	3.4772	0.0005

#### Local autocorrelation

**Figure 4:**
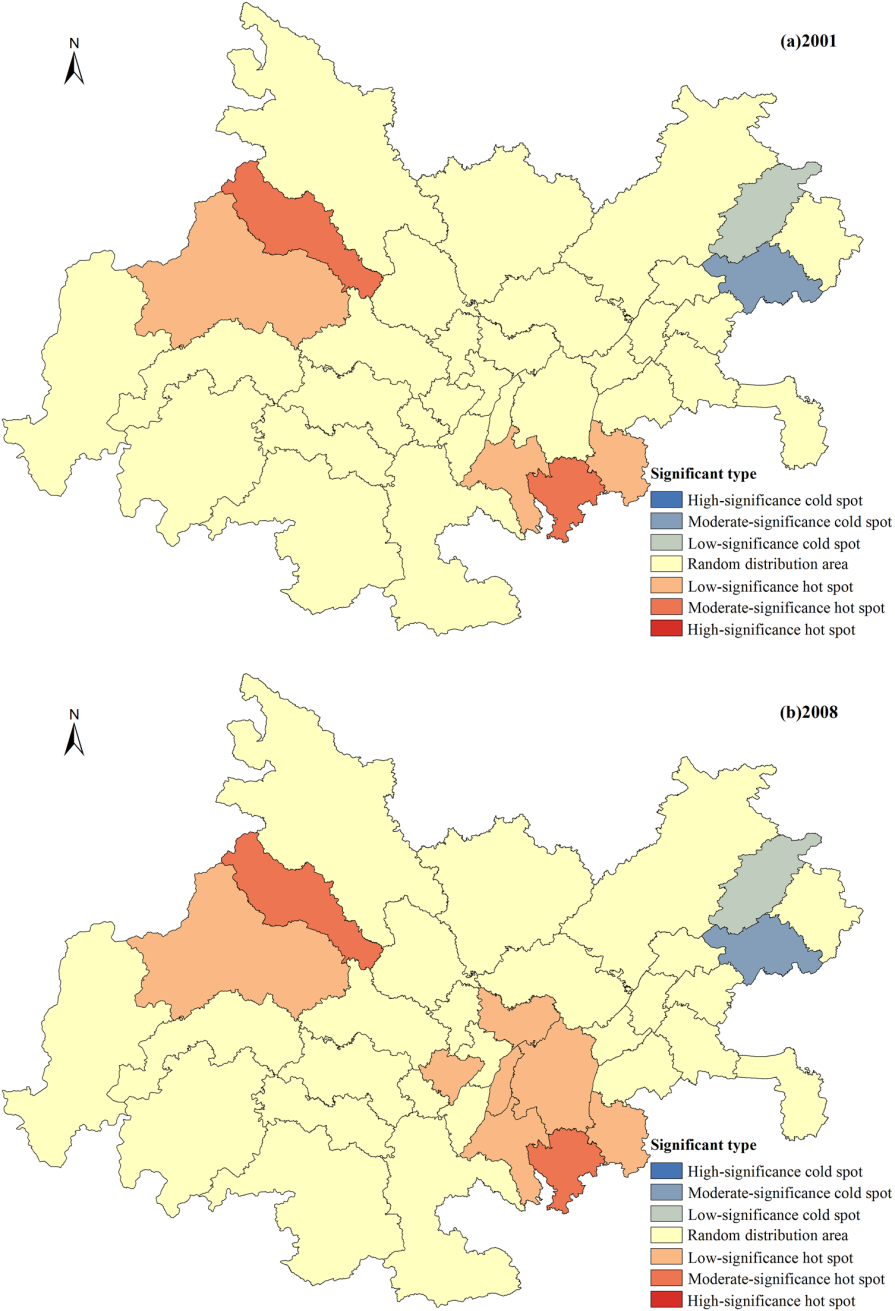

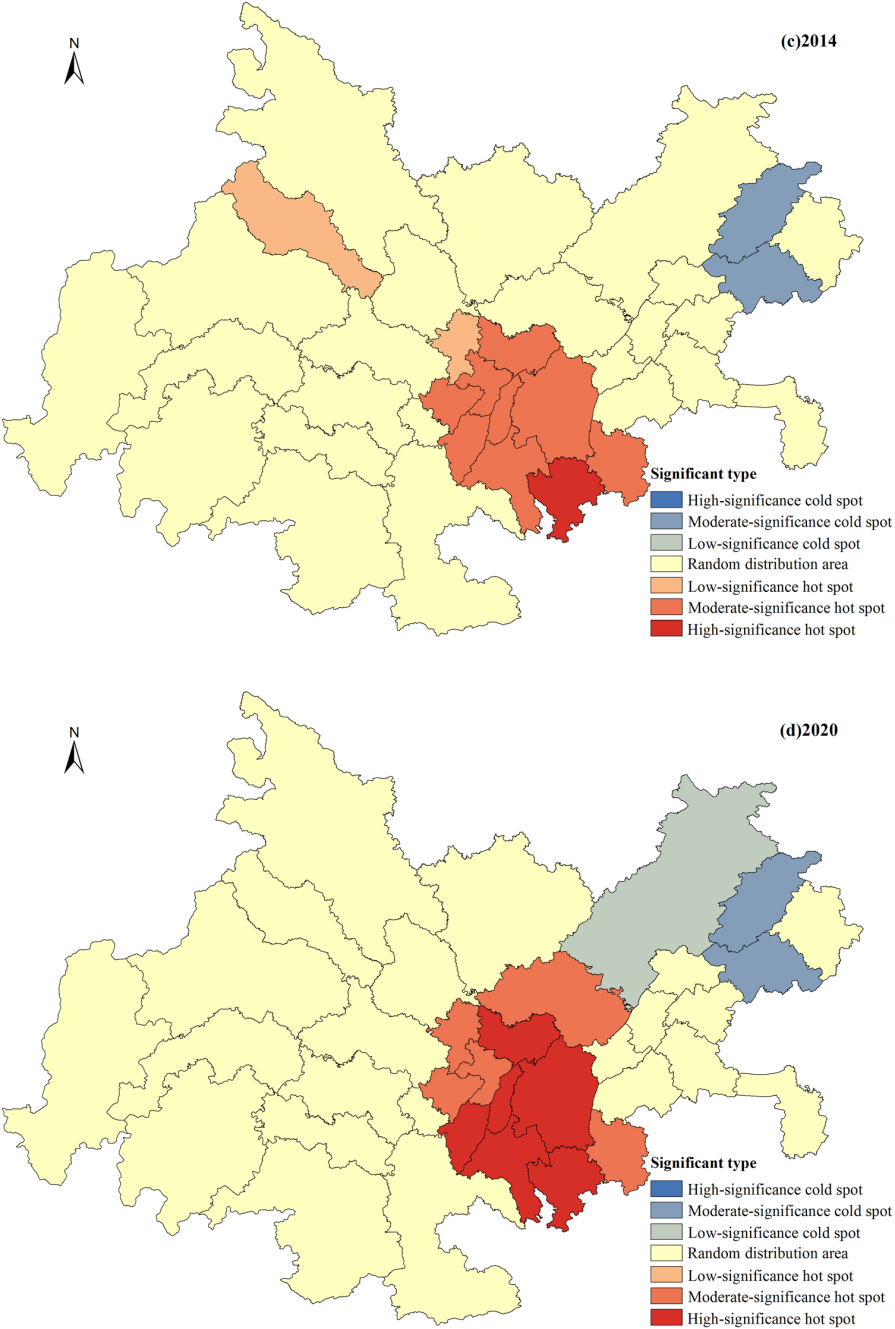
Evolution of hotspots in urbanization and ecological environment coordinated development level (the image was created by the author using ArcGIS 10.6 software).

Global spatial autocorrelation is an analysis of the spatial clustering characteristics of the overall level of coordinated development. To effectively reveal the evolution of local hotspots and identify the contribution of different research units to global autocorrelation, the Getis-Ord G*i values for the urbanization and ecological environment coordinated development level of the Chengdu–Chongqing urban agglomeration in 2001, 2008, 2014, and 2020 were calculated using formula (17). Referring to the relevant literature^57^, these values were classified into seven types: hotspots with high significance, hotspots with medium significance, hotspots with low significance, randomly distributed areas, cold spots with low significance, cold spots with medium significance, and cold spots with high significance (Fig. 4).

1. Overall pattern evolution characteristics. The hotspots and cold spots of the urbanization and ecological environment coordinated development level in the Chengdu–Chongqing urban agglomeration exhibit significant fluctuation characteristics. The distribution range of global hotspots gradually increases and becomes more concentrated, indicating a significant enhancement in the clustering degree of local hotspots. The global cold spots show little change, with a slight expansion in the distribution range in 2020 and an increase in the degree of significance in local cold spots.
2. Hotspot evolution characteristics. The radiation range of hotspots has undergone a transition from a dual-cluster distribution centered on Deyang and Qijiang to a single-cluster distribution centered on Chongqing. The hotspots with high significance have always been located in Chongqing and its surrounding areas. In 2001, the distribution range of hotspots was relatively small, showing a typical “dual-cluster” distribution pattern, with significant-low significant clusters formed by Deyang and Chengdu, as well as by Qijiang, Jiangjin, and Nanchuan, without the presence of highly significant hotspot distribution areas. In 2008, the distribution of hotspots continued to be dominated by the “dual-cluster” pattern, with Dazu standing out as an isolated hotspot with low significance. During this period, the radiation range of hotspots further expanded, with all hotspots showing significant and low significant areas. In 2014, the significant range formed by Deyang and Chengdu began to shrink, while the hotspot cluster centered on Qijiang significantly expanded, and a hotspot with high significance emerged. In 2020, the hotspot pattern completely evolved from a dual-cluster to a single-cluster aggregation form, with the hotspot aggregation area further expanding and the significance intensity significantly increasing. The number of hotspots with high significance also increased from one to six.
3. Cold spot evolution characteristics. The spatial distribution of cold spots has been changing in the eastern region of the urban agglomeration, with little variation in the range and significance level of cold spots. In 2001, 2008, and 2014, the cold spots were located in Wanzhou and Kaizhou, with the cold spots in Kaizhou transitioning from low significance to medium significance. In 2020, the range of cold spots increased, and Dazhou evolved into a cold spot with low significance. Importantly, with the transformation of Dazhou into a cold spot with low significance, a pattern emerged in 2020 where hotspots and cold spots were connected. This feature fully illustrates the significant spatial differences in the coordinated development of the Chengdu–Chongqing urban agglomeration, especially in the southeast‒northeast region of the urban agglomeration, where there is a significant variation in the level of coordinated development between neighboring cities.

### The future evolution trend of urbanization and ecological environment synergistic development

Data related to the synergy degree of each city in the Chengdu–Chongqing urban agglomeration from 2001 to 2015 was inserted into the gray system GM(1,1) model, the synergy degree values from 2016 to 2020 were simulated and predicted, then compared with the actual values, and the MAPE was calculated. The results are shown in Table 6. Except for Qianjiang and Fengdu, the MAPE values of all cities are less than 10%, indicating that the model has highly accurate predictability and can be used for future trend forecasting. The selected forecasting period is from 2021 to 2030, assuming that the control parameter remains as the urbanization subsystem and that there are no significant changes during this period. The predicted results are shown in Table 7.

**Table 6:** Prediction accuracy test of the synergy degree between urbanization and the ecological environment in the Chengdu–Chongqing urban agglomeration.

City	Chengdu	Zigong	Luzhou	Deyang	Mianyang	Suining
MAPE (%)	2.03	4.65	3.78	3.14	4.57	6.99
City	Neijiang	Leshan	Nanchong	Meishan	Yibin	Guang’an
MAPE (%)	5.08	1.49	7.20	4.81	4.55	6.84
City	Dazhou	Ya’an	Ziyang	Chongqing	Wanzhou	Qianjiang
MAPE (%)	6.93	3.39	8.00	3.02	5.54	10.30
City	Fuling	Changshou	Jiangjin	Hechuan	Yongchuan	Nanchuan
MAPE (%)	4.69	5.64	3.93	1.63	2.51	3.80
City	Qijiang	Dazu	Bishan	Tongliang	Tongnan	Rongchang
MAPE (%)	7.03	8.37	5.31	6.92	8.98	5.95
City	Kaizhou	Liangping	Fengdu	Dianjiang	Zhongxian	Yunyang
MAPE (%)	9.78	4.86	10.67	6.52	8.76	7.14

**Table 7:** Future predicted changes in the synergy level of urbanization and ecological environment in the Chengdu–Chongqing urban agglomeration.

City	2021	2022	2023	2024	2025	2026	2027	2028	2029	2030
Chengdu	0.8954	0.9397	0.9862	1.0351	1.0863	1.1401	1.1965	1.2557	1.3179	1.3831
Zigong	0.5349	0.5569	0.5792	0.6018	0.6247	0.6479	0.6715	0.6954	0.7197	0.7443
Luzhou	0.4698	0.4925	0.5156	0.5390	0.5628	0.5869	0.6115	0.6364	0.6617	0.6874
Deyang	0.5657	0.5893	0.6132	0.6375	0.6621	0.6872	0.7125	0.7383	0.7645	0.7910
Mianyang	0.4835	0.5051	0.5270	0.5492	0.5717	0.5946	0.6177	0.6413	0.6651	0.6893
Suining	0.4886	0.5112	0.5342	0.5576	0.5813	0.6053	0.6298	0.6546	0.6797	0.7053
Neijiang	0.4936	0.5143	0.5353	0.5566	0.5781	0.6000	0.6222	0.6447	0.6675	0.6906
Leshan	0.4619	0.4822	0.5027	0.5235	0.5446	0.5659	0.5876	0.6096	0.6319	0.6544
Nanchong	0.4620	0.4840	0.5063	0.5290	0.5520	0.5753	0.5990	0.6231	0.6475	0.6723
Meishan	0.4369	0.4579	0.4792	0.5009	0.5228	0.5451	0.5677	0.5906	0.6139	0.6375
Yibin	0.4687	0.4910	0.5136	0.5366	0.5599	0.5835	0.6075	0.6319	0.6567	0.6818
Guang’an	0.4845	0.5098	0.5354	0.5615	0.5881	0.6151	0.6426	0.6705	0.6989	0.7278
Dazhou	0.3688	0.3861	0.4037	0.4215	0.4395	0.4578	0.4762	0.4950	0.5139	0.5331
Ya’an	0.3996	0.4168	0.4341	0.4517	0.4695	0.4875	0.5057	0.5241	0.5428	0.5617
Ziyang	0.4420	0.4634	0.4852	0.5072	0.5296	0.5524	0.5754	0.5988	0.6226	0.6467
Chongqing	0.9333	0.9692	1.0056	1.0428	1.0806	1.1192	1.1584	1.1984	1.2391	1.2806
Wanzhou	0.5499	0.5720	0.5944	0.6172	0.6402	0.6636	0.6873	0.7114	0.7358	0.7605
Qianjiang	0.5201	0.5476	0.5755	0.6039	0.6328	0.6622	0.6922	0.7227	0.7537	0.7854
Fulin	0.7034	0.7369	0.7711	0.8059	0.8414	0.8777	0.9146	0.9523	0.9907	1.0298
Changshou	0.7030	0.7368	0.7713	0.8064	0.8423	0.8788	0.9161	0.9541	0.9929	1.0324
Jiangjin	0.5937	0.6210	0.6488	0.6771	0.7059	0.7351	0.7649	0.7951	0.8259	0.8572
Hechuan	0.5696	0.5952	0.6213	0.6477	0.6747	0.7020	0.7298	0.7581	0.7868	0.8160
Yongchuan	0.7093	0.7437	0.7788	0.8146	0.8512	0.8884	0.9265	0.9652	1.0048	1.0452
Nanchuan	0.4661	0.4872	0.5086	0.5303	0.5523	0.5747	0.5973	0.6203	0.6437	0.6673
Qijiang	0.5891	0.6202	0.6518	0.6841	0.7171	0.7506	0.7848	0.8197	0.8553	0.8916
Dazu	0.6395	0.6756	0.7125	0.7501	0.7886	0.8280	0.8682	0.9093	0.9513	0.9942
Bishan	0.7165	0.7536	0.7916	0.8303	0.8699	0.9104	0.9517	0.9939	1.0371	1.0812
Tongliang	0.6570	0.6912	0.7261	0.7618	0.7982	0.8353	0.8733	0.9120	0.9514	0.9918
Tongnan	0.5623	0.5932	0.6246	0.6567	0.6894	0.7227	0.7567	0.7914	0.8268	0.8628
Rongchang	0.6652	0.7006	0.7367	0.7735	0.8112	0.8497	0.8889	0.9291	0.9700	1.0118
Kaizhou	0.4107	0.4312	0.4520	0.4731	0.4945	0.5163	0.5383	0.5607	0.5834	0.6064
Liangping	0.4655	0.4893	0.5134	0.5380	0.5629	0.5883	0.6140	0.6402	0.6668	0.6939
Fengdu	0.4312	0.4553	0.4799	0.5049	0.5303	0.5562	0.5825	0.6092	0.6364	0.6640
Dianjiang	0.4924	0.5185	0.5451	0.5721	0.5997	0.6277	0.6562	0.6852	0.7147	0.7447
Zhongxian	0.4539	0.4780	0.5026	0.5276	0.5529	0.5787	0.6050	0.6316	0.6587	0.6863
Yunyang	0.3793	0.3998	0.4206	0.4417	0.4631	0.4848	0.5069	0.5293	0.5520	0.5751

The prediction results indicate a positive future trend in the coordinated development of urbanization and the ecological environment in the Chengdu–Chongqing urban agglomeration. From 2021 to 2030, the average synergy development index of the urban agglomeration increases from 0.5235 to 0.8023, reaching a high level of coordinated development overall. The synergy development index of each city continues to increase year by year, but the disparity in synergy levels further widens. By 2030, the synergy development index of Dazhou is the lowest at 0.5331, which is less than half that of Chengdu. Table 5 shows that in 2023, the synergy degrees of Chengdu and Chongqing increase to 0.9862 and 1.0056, respectively, surpassing the steady-state value of the system (0.9749). By 2030, Chengdu, Chongqing, Fuling, Changshou, Yongchuan, Dazu, Bishan, Tongliang, and Rongchang, a total of 9 cities, have synergy degrees exceeding the steady-state value. This phenomenon indicates that with the evolution of the synergy between urbanization and the ecological environment, the synergistic effects of these cities are becoming stronger. They have transitioned from an ordered state to a new disordered state, breaking out of the existing synergy system and entering a higher stage of coordinated development.

## Discussion

### Construction of the urbanization and ecological environment system evaluation index system

The construction of the index system is an important foundation for exploring the coordinated relationship between urbanization and the ecological environment. Urbanization is a complex dynamic process that involves changes in factors such as population, industry, society, space, and ecology. The ecological environment is a collection of interactions between resources, the environment, and organisms. To fully reflect the urbanization process and the content of the ecological environment, comprehensive index systems are typically constructed. Researchers usually choose reasonable evaluation indicators based on a “standard” framework, among which PESS and PSR are currently the most classic framework models for constructing urbanization and ecological environment index systems ^53,54^. Based on this, a comprehensive evaluation index system for urbanization systems and ecological environment systems is constructed in this paper. This section discusses and explains some of the indicators. In terms of spatial urbanization, the area of urban built-up areas is an important consideration. Some scholars directly use the total area of urban built-up areas as an indicator of spatial urbanization^55^, while others choose per capita urban built-up area^2^. Since there are differences in the areas of different research units, the total area of urban built-up areas cannot reflect these spatial size differences. At the same time, due to the regulations of urban planning standards, there is not much difference in per capita built-up area among cities, making it difficult to measure spatial urbanization. Urban area density is calculated by dividing the total area of urban built-up areas within the research unit by the total area of the research unit. This process reflects the size of urban built-up areas per unit area and more accurately reflects the urban spatial expansion of different-sized research units. Therefore, urban area density is selected as one of the indicators to measure spatial urbanization. In terms of social urbanization, education indicators are a good choice. However, the existing statistics on primary and secondary school education generally do not distinguish between urban and rural areas, making it difficult to evaluate the level of social urbanization. Typically, some scholars use the number of university students as an education indicator^34^. However, according to the actual situation of the research area in this paper, universities are mainly concentrated in Chengdu and Chongqing, and most research units do not have universities. If the number of university students is included as an education indicator in the urbanization evaluation index system, it may lead to abnormal evaluation results. Therefore, this paper does not consider education indicators. In addition, due to data limitations and difficulties in quantifying variables, some indicators have not been included, such as economic trade and technology indicators that describe urbanization, as well as indicators that describe the ecological environment, such as air quality and soil pollution. In summary, based on previous research, considering the actual situation of the research area and the availability of data, this study has carefully examined and compared and comprehensively constructed an evaluation index system for urbanization and the ecological environment. This index system will be further optimized in future studies of the same type.

### Reflection on the application of the Haken model in the study of the synergistic relationship between urbanization and the ecological environment

The introduction of the theory of synergy into the study of urbanization and ecological environment relationships can enlighten the theory of human-earth systems and help solve practical problems related to these relationships. As an important component of the theory of synergy, the Haken model determines whether the various parameters in the system satisfy the adiabatic approximation assumption and solves for the potential function. It constructs the evolution equation, thus obtaining the order parameter equation and the evolution equation group of the system. This ability to solve the evolution process of the equation group can effectively support the study of the self-organizing and synergistic evolution process of complex systems, which is highly persuasive^56^. This model has achieved good results in analyzing the mechanism and coordinated development of urbanization and the ecological environment. However, there are also several points worth discussing and considering: (1) According to the theory of synergy, the interaction of various factors within the system and the transformation from disorder to order are premised on the assumption that changes in the external environment will not affect the occurrence of mutations or large-scale changes in various parts of the system^57^. An urban agglomeration is a giant, complex system, and the complex urbanization and ecological environment system is just one part of it. When other systems within the urban agglomeration undergo mutations, the interactions of this complex system with its internal subsystems and elements may be affected, and the synergistic effect will stop responding. (2) The Haken model is based on the order parameter evolution equation, which uses the potential function; it requires the system to have characteristics of openness, nonlinearity, and nonequilibrium. However, according to the principle of order parameter, the order parameter appears only when the subsystems are interrelated, and one or two of the subsystems dominate in the motion of the complex system. This model is not applicable in systems where the order parameters cannot be found^58^. If the interaction between the urbanization system and the ecological environment system is small or maintains a relatively consistent evolutionary relationship, it may not be possible to determine the order parameters of the complex system evolution, and the Haken model is no longer applicable. (3) As the complex system evolves, the order parameter also changes. Therefore, in the study of the long-term evolutionary relationship between urbanization and the ecological environment, to better identify the order parameters of system evolution and their evolution process, it is necessary to divide the research period into multiple stages and conduct multistage studies via the Haken model. This has been verified in other fields of research^41,49^.

### Policy impact and recommendations

Our research findings have important implications for policy making and promoting local development. (1) In terms of urbanization, different regions should adopt different urban development models to promote high-quality and balanced regional urbanization. The research results show that although the synergy between urbanization and the ecological environment is gradually increasing, most cities are still at a relatively low level. At the same time, there are significant spatial differences in synergy, which is consistent with the law of urbanization development. Therefore, to improve the level of synergy between urbanization and the ecological environment, the key lies in balancing the promotion of urbanization without harming the ecological environment. Different economic development and urbanization models should be adopted for cities in different regions with different resource endowments. For the two core cities of Chengdu and Chongqing, while their core status should be strengthened, their radiation effects should be enhanced to gradually promote the outward migration of labor-intensive industries and other noncore functions in an orderly manner, thereby driving the coordinated development of surrounding cities. For central cities in areas such as Mianyang, Yibin, Nanchong, and Wanzhou, interaction and cooperation with Chengdu and Chongqing should be strengthened to give full play to their regional radiation and driving effects and to improve the overall functions and service levels of the cities. For mountainous cities surrounding urban clusters such as Ya’an, Dazhou, Kaizhou, Yunyang, and Qianjiang, infrastructure construction should be intensified to promote cooperation and circulation in transportation, logistics, information, and other aspects with regional central cities. Furthermore, the development of characteristic advantageous industries should be strengthened to enhance urban vitality and attractiveness. (2) In terms of the ecological environment, efforts should be made to continuously optimize the ecological environment and enhance its carrying capacity. First, coordinated efforts should be made to build and protect the regional ecological environment. The planning, construction, and protection of ecological space should be carried out simultaneously, and the ecological system of key ecological functional areas should be nurtured and restored. For cities with relatively low forest coverage in the central part of urban agglomerations, such as Ziyang, Neijiang, Zigong, Hechuan, and Rongchang, tree planting and afforestation should be strengthened to increase forest coverage and enhance the stability and resistance of the ecological system. For remote mountainous cities such as Qianjiang, Yunyang, Ya’an, and Dazhou, environmental protection mechanisms and protection systems should be established, residents’ environmental awareness should be enhanced, and environmental protection work should be promoted effectively. Second, coordinated efforts should be made to address cross-border environmental pollution in the region. The formulation of technical specifications and initiation of joint actions can collectively address pollution issues such as air, water, soil, hazardous waste, and noise.

### Limitations and future directions

This study examines the synergistic development relationship between urbanization and the ecological environment based on administrative boundaries. However, the administrative divisions within the Chengdu–Chongqing urban agglomeration are complex, and there are significant differences among the basic units. The urban agglomeration includes two provincial-level administrative regions, Sichuan Province and Chongqing Municipality. Sichuan Province follows a provincial-city-county administrative system, while Chongqing Municipality operates under a direct-controlled municipality-district/county system. In terms of administrative management, the districts/counties in Chongqing Municipality are at the same level as the cities in Sichuan Province, but in terms of geographical scope, they are equivalent to a county within Sichuan Province. This administrative division system results in significant differences in the size of the basic units in this study, which may lead to substantial variations in related development indicators. A comparison of the results across units may be affected by these differences. Therefore, in future research, it is necessary to consider a more scientifically based division of research units, such as ignoring the administrative boundaries and instead studying the synergistic effects between urbanization and the ecological environment at the grid scale. This approach can better reflect the mutual flow and integration of various elements within the urban agglomeration across administrative regions. In addition, the optimization of the indicator system for urbanization and ecological environment evaluation is worth further attention in the future. Urbanization and the ecological environment cover wide areas; furthermore, numerous evaluation indicators are involved in studies such as these. Hence, the current indicator system often carries strong subjectivity. More scientific and objective methods for indicator system construction in the future should be explored to enhance the accuracy and reliability of evaluation results.

## Conclusions

While the relationship between urban agglomerations and the urbanization-ecological environment nexus has garnered global attention, previous research has paid little attention to the synergistic effects between urbanization and the ecological environment. This study fills this gap by employing the theory of synergy and the Haken model to empirically investigate the synergistic effects between urbanization and the ecological environment in the Chengdu–Chongqing urban agglomeration. The findings provide policy implications for the sustainable development of the Chengdu–Chongqing urban agglomeration and serve as a theoretical reference for research on the synergetic development of urbanization and the ecological environment in other urban agglomerations.

1. Both order parameters and control parameters influence the synergetic development between urbanization and the ecological environment. The order parameters determine the direction and path of system evolution, while the control parameters reflect the system’s evolutionary behavior. In the urbanization and ecological environment system of the Chengdu–Chongqing urban agglomeration, the order parameter is UR, and the control variables *a* and *b* indicate that the ecological environment has a negative impact on urbanization development, while urbanization development hinders the improvement of the ecological environment. The control variables *γ*_1_ and *γ*_2_ indicate that urbanization has established a positive feedback mechanism to promote the orderly evolution of the system, while the ecological environment subsystem has established a negative feedback mechanism to enhance orderliness. The absolute values of the four control variables are relatively small, indicating that the degree of influence between the two subsystems is still relatively light, and the current system is still in a stage of low-level order development.
2. The urbanization and ecological environment synergetic development level of the Chengdu–Chongqing urban agglomeration is relatively low, with significant spatial differences. From 2001 to 2020, the degree of synergy between urbanization and the ecological environment in the Chengdu–Chongqing urban agglomeration ranged from 0.1419 to 0.5235, indicating a generally low level of synergetic development but with a gradual upward trend. The types of synergetic development mainly underwent a transition from low-level synergy to medium–low synergy and then to medium synergy, with fewer instances of medium–high and high synergy. The analysis reveals a leading region of synergetic development in the northwest and southeast, centered around Chengdu and Chongqing, as well as lagging regions of synergetic development in the eastern cities of Dazhou, Kaizhou, and Yunyang. Spatial autocorrelation analysis shows that since 2014, the spatial distribution of the level of synergy between urbanization and the ecological environment has gradually exhibited spatial autocorrelation, with significant high-value and low-value agglomeration characteristics. Moreover, over time, this spatial autocorrelation and agglomeration tendency have strengthened. Local autocorrelation results indicate the presence of hotspots and cold spots at each level of synergetic development between urbanization and the ecological environment in the Chengdu–Chongqing urban agglomeration. The distribution range of global hotspots has gradually increased and become more concentrated, while the degree of clustering in local hotspots has significantly increased. The distribution of global cold spots has remained relatively stable, with a slight increase in the degree of clustering in local cold spots.
3. The level of synergetic development between urbanization and the ecological environment in the Chengdu–Chongqing urban agglomeration will gradually increase in the future. When the degree of synergy exceeds the steady-state value of the system, the city will enter a higher-level system and begin a new phase of synergetic development. The gray prediction GM (1,1) results indicate a positive future trend in the synergetic development of urbanization and the ecological environment in the Chengdu–Chongqing urban agglomeration over the next 10 years. The average index of synergetic development in the urban agglomeration is projected to increase from 0.5235 to 0.8023, reaching a high level of synergetic development overall. The index of synergetic development for each city continues to increase year by year, but the disparity in the level of synergy further widens. As time progresses, Chengdu and Chongqing are expected to surpass the system’s steady-state value (0.9749) in terms of the degree of synergy between urbanization and the ecological environment. By 2030, nine cities, Chengdu, Chongqing, Fuling, Changshou, Yongchuan, Dazu, Bishan, Tongliang, and Rongchang, are projected to have a degree of synergy exceeding the steady-state value, transitioning from an ordered state to a new disordered state. This breakthrough in the existing synergetic system signifies entry into a higher-level phase of synergetic development.

## Data Availability

Upon publication of the paper, the datasets used and analyzed during the study are available from the corresponding author upon reasonable request.
